# Coffee-Leaves as a Substitute for Tea

**Published:** 1853-08

**Authors:** 


*Coffee-Leaves as a Substitute for Tea.*—From the *Overland Singapore Free Press*, published Jan. 3, 1853, we extract the following letter signed "*An old Sumatran*," upon the use of coffee leaves for the preparation of a beverage in the island of Sumatra. We briefly alluded in the *Pharmaceutical Journal* for June, 1852 (Vol. xi., p. 578,) to a project for employing coffe-leaves in this country as a substitute for tea.

"In the *Singapore Free Press*, of the 17th of September last, are extracts from the *Colombo Observer*, by which it appears a patent has been taken out by Dr. Gardner (known to us by his travels in South America*) for preparing the coffe-leaf in a manner to afford a beverage like tea, that is, by infusion, 'forming an agreeable, refreshing, and nutritive article of diet.'

"It may be interesting to Dr. Gardner, his friends, and the public in general to learn, that an infusion of the coffee-leaf is an article of universal consumption amongst the natives of this part of Sumatra; wherever coffee is

*It is Dr. John Gardner of London who exhibited prepared coffee-leavee at the Great exhibition of 1851. Mr. George Gardner, late superintendent of the Botanical Garden at Peradenia, Ceylon, and author of *Travels in the Interior of Brazil*, died in Ceylon in March, 1849. —Ed. *Pharm. Journ.*

grown the leaf has become one of the very few necessaries of life which the natives regard as indispensable.

"The coffee plant in a congenial soil and climate exhibits great luxuriance in its foliage, throwing out abundance of suckers and lateral stems especially when from any cause the main stem is thrown out of the perpendicular, to which it is very liable from its great superincumbent weight compared with the hold of its roots in the ground. The native planters, availing themselves of this propensity, often give the plant a considerable inclination not only to increase the foliage, but to obtain new fruit-bearing stems when the old ones become unproductive. It is also found desirable to limit the height of the plant by lopping off the top, to increase the produce and facilitate collecting it, and fresh sprouts in abundance are the certain consequence. These are so many causes of the development of a vegetation which becomes injurious to the quantity of the fruit or berry unless removed; and where this superabundant foliage can be converted into an article of consumption, as hitherto the case in Sumatra, the culture must become the more profitable, and it is clearly the interest of the planters of Ceylon to respond to the call of Dr. Gardner, and by supplying the leaf on reasonable terms, to assist in creating a demand for an article they have in abundance, and which for the want of that demand is of no value to them. It ought to be mentioned also, that the leaves which become ripe and yellow on the tree and fall off in the course of nature, contain the largest portion of extract and make the richest infusion, and I have no doubt, should the coffee-leaf ever come into general use, the ripe leaf will be collected with as much care as the ripe fruit.

"The mode of preparation by the natives is thus:—The ends of the branches and suckers with the leaves on, are taken from the tree and broken into lengths of from twelve to eighteen inches. These are arranged in the split of a stick or small bamboo, side by side, forming a truss in such a manner, that the leaves all appear on one side and the stalks on the other, the object of which is to secure equal roasting, the stalks being thus exposed to the fire together and the leaves together. The slit being tied up in two or three places, and a part of the stick or bamboo left as a handle, the truss is held over a fire without smoke, and kept moving about so as to roast the whole equally without burning, on the success of which operation the quality and flavor of the article much depends. When successfully roasted the raw vegetable taste is entirely dissipated, which is not the case if insufficiently done. When singed or overdone, the extract is destroyed and the aroma lost. When the fire is smoky, the flavor varies with the nature of the smoke. The stalks are roasted equally with the leaves, and are said to add fully as much to the strength ●f the infusion. By roasting, the whole becomes brittle, and is reduced to a coarse powder by rubbing between the hands. In this state it is ready for use, and the general mode of preparing the beverage is by infusion, as in the case of common tea.

"If the testimony of one who has been long personally accustomed to the use of an infusion of the coffee-leaf thus prepared, can be of any avail in reccommending the article to public notice, I freely offer mine in support of all that which Dr. Gardner's patent claims for it, viz., 'as forming an agreeable, refreshing, and nutritive article of diet.' While I find the use of infusion of the *berry* for a few days invariably to produce on me, as on many others, the effects of nervousness and bilious obstruction, I drink a strong infusion of the *leaf* daily with evident benefit to my health and strength. As a restorative on exhaustion from the severities of labor or of the weather, from heat to cold, or long exposure to rain, I know nothing superior to it. It has also the advantage of being a powerful disinfectant, so far as neutralizing fœtidity goes, and a solvent of the viscid fluids which obstruct the circulation, often to the extent of becoming laxative if taken in extra quantity. Of its nutritive power, no proof can be stronger than that it suspends hunger and enables the laboring man to pursue his work for hours after he wonld be otherwise unable. That it would soon become a most valuable article of diet among the laboring classes, and on ship-board particularly, if once brought into use, there can be no doubt. The coffee-tree can be grown to advantage for the leaf in the lowlands of every tropical country where the soil is sufficiently fertile, whilst it requires soil and climate to produce the fruit.

"Nothing appears in the *Free Press* on the mode of its preparation by Dr. Gardner, but I should think if roasted and pulverized and packed in air-tight cases like tea, it would retain its strength and bear transporting to every part of the world; and as it soon fixes itself more strongly than either tea or coffee in the taste, it would soon become a more absolute necessary of life than either of those articles. In fact, I am acquainted with no tropical production capable of being rendered so great a blessing to mankind as the coffe leaf; and as it would tend materially to the desuetude of ardent spirits and strong drinks, its introduction ought to have the support of every friend to the moral and material welfare of society."—*Amer. Jour. of Pharmacy.*

